# Conservation and divergence between cytoplasmic and muscle-specific actin capping proteins: insights from the crystal structure of cytoplasmic Cap32/34 from *Dictyostelium discoideum*

**DOI:** 10.1186/1472-6807-12-12

**Published:** 2012-06-01

**Authors:** Christian Eckert, Agnieszka Goretzki, Maria Faberova, Martin Kollmar

**Affiliations:** 1 Abteilung NMR basierte Strukturbiologie, Max-Planck-Institut für Biophysikalische Chemie, Am Fassberg 11, D-37077, Göttingen, Germany

**Keywords:** Capping protein, Actin-binding, *Dictyostelium discoideum*, Structural flexibility, Cap32/34, CapZ

## Abstract

**Background:**

Capping protein (CP), also known as CapZ in muscle cells and Cap32/34 in *Dictyostelium discoideum*, plays a major role in regulating actin filament dynamics. CP is a ubiquitously expressed heterodimer comprising an α- and β-subunit. It tightly binds to the fast growing end of actin filaments, thereby functioning as a “cap” by blocking the addition and loss of actin subunits. Vertebrates contain two somatic variants of CP, one being primarily found at the cell periphery of non-muscle tissues while the other is mainly localized at the Z-discs of skeletal muscles.

**Results:**

To elucidate structural and functional differences between cytoplasmic and sarcomercic CP variants, we have solved the atomic structure of Cap32/34 (32 = β- and 34 = α-subunit) from the cellular slime mold *Dictyostelium* at 2.2 Å resolution and compared it to that of chicken muscle CapZ. The two homologs display a similar overall arrangement including the attached α-subunit C-terminus (α-tentacle) and the flexible β-tentacle. Nevertheless, the structures exhibit marked differences suggesting considerable structural flexibility within the α-subunit. In the α-subunit we observed a bending motion of the β-sheet region located opposite to the position of the C-terminal β-tentacle towards the antiparallel helices that interconnect the heterodimer. Recently, a two domain twisting attributed mainly to the β-subunit has been reported. At the hinge of these two domains Cap32/34 contains an elongated and highly flexible loop, which has been reported to be important for the interaction of cytoplasmic CP with actin and might contribute to the more dynamic actin-binding of cytoplasmic compared to sarcomeric CP (CapZ).

**Conclusions:**

The structure of Cap32/34 from *Dictyostelium discoideum* allowed a detailed analysis and comparison between the cytoplasmic and sarcomeric variants of CP. Significant structural flexibility could particularly be found within the α-subunit, a loop region in the β-subunit, and the surface of the α-globule where the amino acid differences between the cytoplasmic and sarcomeric mammalian CP are located. Hence, the crystal structure of Cap32/34 raises the possibility of different binding behaviours of the CP variants toward the barbed end of actin filaments, a feature, which might have arisen from adaptation to different environments.

## Background

Actin is a key component in all eukaryotic cells and plays an essential role in a wide range of cellular processes, such as migration, endocytosis, cytokinesis and generation of contraction [1-4]. Actin monomers (G-actin) are able to polymerize into filamentous actin (F-actin) resulting in polar helical structures [5]. The two ends of the filament exhibit distinct biochemical properties and are differentiated as “barbed” and “pointed”, so named after the arrowhead appearance when filaments are decorated with myosin S1 [6]. Filament barbed ends dominate the dynamics of filament assembly due to higher association and dissociation rates for actin monomers compared to the pointed ends [3,7,8]. Furthermore, since the filament barbed end is preferred for actin monomer addition, whereas net disassembly is favoured at its counterpart, it is being referred to as the fast growing end (pointed end = slow growing end).

In living cells the actin cytoskeleton is in a state of rapid dynamics. Remodelling of the actin cytoskeleton is crucial in terms of inducing changes in cell shape, motility and adhesion and requires strict regulation, both temporally and spatially, thus enabling the cell to function in a controlled manner [4,9]. This is achieved by a vast number of specialized proteins that bind to actin, thereby modulating actin filament organization and turnover in response to the changing needs of the cell [10,11]. Actin-binding proteins are able to fulfil a large variety of tasks including the control of actin assembly and disassembly as well as regulating filament branching and bundling to help arrange actin filaments into higher order structures [12]. They can be categorized into proteins which bind to actin monomers, filamentous actin or both [10]. While actin monomer binding proteins control the amount and availability of monomers for polymerization, proteins that bind filamentous actin are involved, among others, in barbed and pointed end capping, filament severing, and filament crosslinking.

Capping protein (CP) is an F-actin binding protein and blocks actin filament elongation and turnover by preventing the addition of new monomers at the fast growing end [11]. Binding of CP to actin filaments occurs with high affinity (K_d_ < 1 n*M*) and 1:1 stoichiometry. Two major variants of CP have been determined: a cytoplasmic form that is also termed Cap32/34 (32 = β- and 34 = α-subunit; [13]) and an isoform found in the Z-discs of skeletal muscles that is often called CapZ [14,15]. CP is a heterodimeric protein composed of an α- and a β-subunit, both having molecular masses in the range of 30–36 kDa. The protein is expressed in all eukaryotic organisms and the subunits exhibit high sequence similarity across the eukaryotic tree of life [11].

Vertebrates usually express three conserved isoforms of each of the α- and β-subunit [16-18] as opposed to invertebrates, plants, and lower eukaryotes, which in general contain single isoforms of each subunit. The vertebrate α-subunit isoforms are encoded by different genes [19], whereas the β-subunits arise by alternative splicing from a single gene [16,17]. One isoform of both the α- and β-subunits is specifically expressed in germ cells (α3, β3), while the remaining ones (α1, α2 and β1, β2) are somatically expressed at varying ratios in different cell types and tissues [19]. β1 is the predominant isoform in muscle cells. In contrast, β2 is mainly expressed in non-muscle tissues [17]. The β isoforms are not able to rescue each others’ function and are thus believed to fulfil different biochemical and cellular tasks [20]. On the other hand, there is little indication of specific functions for the α isoforms [11].

Vertebrates contain two somatic variants of CP. The sarcomeric variant, which is being referred to as CapZ throughout this manuscript, includes the β1 isoform and is positioned at the Z-discs of striated muscles [14]. CapZ is proposed to help attaching actin filament barbed ends to the Z-discs and to prevent the thin filaments from growing into the adjacent sarcomere, thus serving as a key element in thin filament assembly and regulation within the Z-disc [11]. By contrast, the cytoplasmic variant, which comprises the β2 isoform, is found at the contact sites of actin with membranes [21], where it is believed to play an essential role in the dendritic nucleation model [22]. In this model activation of the Arp2/3 complex results in a branched network of actin filaments thereby generating new barbed ends, which are primarily oriented towards the cell membrane. As actin subunits are added to the newly created filament ends the membrane is pushed forward [12]. By capping these ends over time, the growing filaments are kept short and branched, which stabilizes the filament network and sustains the propulsive force for leading edge elongation of migrating cells [1]. In addition, actin assembly is restricted to the new barbed ends near the plasma membrane [23], thus enabling rapid and directed extension of the cell front.

Several molecules are able to modulate the barbed end capping activity of CP by either binding directly to the protein or through association with filament barbed ends and thereby inhibiting CP from binding. Polyphosphoinositides (PPIs), such as phosphatidylinositol-4,5-bisphosphate (PIP_2_) [24-26] and the proteins CARMIL [27] and V-1 [28] were found to directly associate with CP and to inhibit its capping activity. The crystal structures of CapZ (chicken α1/β1) in complex with CARMIL and V-1, respectively, were recently reported [29,30]. However, to date no high resolution structure of CP bound to PIP_2_ exists. One possible role of PIP_2_, an important component of the plasma membrane and one of the most potent signalling lipids, might be to facilitate membrane movement of highly motile cells, such as those of *Dictyostelium discoideum*, through inhibition of actin filament capping by CP near the membrane [24], thus allowing rapid protrusion of the cell edge. Computational docking studies predict that PIP_2_ interacts with a set of three highly conserved basic residues in close proximity to the α-subunit’s C-terminus [25]. Two of these basic residues are critical for actin filament capping [31]. Such an interaction would therefore prevent for steric reasons the ability of CP to associate with the actin filament.

The crystal structure of CapZ (chicken α1/β1) [32] has provided valuable insight into the atomic architecture of CP found at the Z-discs of skeletal muscles. However, until now a high-resolution structure of the cytoplasmic variant is not available. By characterizing the atomic structure of Cap32/34 from the cellular slime mold *Dictyostelium discoideum* as a model for cytoplasmic CP and comparing it to that of CapZ, we aimed to elucidate structural and functional differences between the two CP isoforms. This allowed us to shed light on potential interaction sites with muscle and non-muscle specific components, respectively.

## Methods

### Protein isolation and purification

The *Dictyostelium discoideum* Cap32 and Cap34 subunits were co-expressed in *Escherichia coli* using pETmD1-mako, an expression vector, which allows simultaneous expression of the two subunits. This vector was built on pETDuet™-1 (Novagen) by replacing the second MCS by the MCS of pDXA-mako [33] for easy shuttling of genes between bacterial and *Dictyostelium* expression vectors. A full-length cDNA clone for Cap34 was obtained from the Japanese *Dictyostelium* cDNA project (clone VFM643; [34]). Cap32 was assembled from two overlapping cDNA clones, SSA656 and SSJ183 [35,36]. Cap32 and Cap34 were PCR-amplified applying the Expand High Fidelity PCR System (Roche) from cDNAs using primer A (5′-GGTTATGTACAAGGTACAGAAAAGCAATTAAGTTGTTGTCTCG -3′; Cap32, forward, *Bsr*GI site underlined) and primer B (5′- CCGACGCGTACTACCAGCAAGATTTACTTTACCAG -3′; Cap32, reverse, *Mlu*I site underlined) for Cap32, and primer C (5′- CCGCCATGGCCTCAAATCAAGAATCGTTCAAATC-3′; Cap34, forward, *Nco*I site underlined) and primer D (5′- CCGACGCGTAAGCTTTTTTTATTTTCATTGGCAATTTTGAAGTTTTTG -3′; Cap34, reverse, *Hind*III site underlined) for Cap34, respectively. The PCR products were digested and subsequently ligated into pETmD1-mako. Thereby, the coding sequence of Cap32 is fused to an N-terminal 8xHis tag.

The plasmid was transformed into *Escherichia coli* BL21-CodonPlus(DE3)-RIL strain cells (Stratagene), which were cultured in LB broth containing 80 μg ml^−1^ ampicillin at 37°C until an OD(600 nm) of 0.6–0.8 was reached. Protein expression was induced at 22°C by the addition of IPTG to a final concentration of 0.1 mM and the cell culture was allowed to grow overnight. The cells were harvested by centrifugation at 6,000 X g for 15 min and the cell pellets were stored at −20°C. For protein purification the cell pellets were resuspended in 20 mM Hepes pH 7.3, 50 mM NaCl, 0.1 mM PMSF, and 1 mM DTT supplemented with EDTA-free protease-inhibitor-cocktail (Roche) and disrupted on ice by sonication. The lysate was centrifuged at 37,000 X g for 40 min to remove cellular debris.

The supernatant was sterile filtered through an Ultrafree-MC GV 0.22 μm syringe filter (Millipore) before application onto a 10 ml column of Ni-NTA superflow resin (Qiagen) pre-equilibrated with IMAC buffer (buffer A: 50 mM Hepes pH 7.3, 30 mM KAc). The column was extensively washed first with buffer A and then with 50 mM Hepes pH 7.3, 300 mM KAc (buffer B) to remove non-specifically bound proteins. The third washing step was performed with buffer A including 40 mM imidazole pH 7.3, and finally Cap32/34 was eluted from the column using a linear gradient of 40–500 mM imidazole pH 7.3 in buffer A. Fractions containing the target protein were pooled and dialyzed against 20 mM Hepes pH 7.3, 100 mM NaCl, 0,5 mM EDTA, 0.1 mM EGTA, and 1 mM MgAc. After protein concentration using a Vivaspin 6 30 k (GE Healthcare), Cap32/34 was further purified by size-exclusion chromatography on a HiLoad 16/60 Superdex 200 column (GE Healthcare) equilibrated and run with 20 mM Hepes pH 7.3, 100 mM NaCl, 0,5 mM EDTA, 0.1 mM EGTA, and 1 mM MgAc.

After checking the protein purity by SDS-PAGE, the sample was concentrated to 8 mg ml^−1^ with a Vivaspin 6 30 k (GE Healthcare) and supplemented with sucrose to a final concentration of 3% (*w*/*v*). The protein was then divided into 50 μl aliquots in thin-walled PCR tubes, flash-cooled in liquid nitrogen, and stored at −80°C. A total of ~20 mg pure Cap32/34 was obtained from 1 L cell culture.

### Crystallization, data collection, and processing

Crystallization trials were performed using hanging-drop vapor diffusion with standard sparse-matrix screens. Drops were prepared by manually dispensing 2 μl of protein solution with 2 μl reservoir solution and equilibrated against 400 μl reservoir solution in 24-well VDX plates (Hampton Research). Initial crystals formed in 100 mM Hepes pH 7.5, 20% (*w*/*v*) PEG 8000 at 20°C and grew to typical dimensions of 10 × 10 × 120 μm within 4–6 d. Subsequently, crystals were optimized by micro-seeding. The best diffracting crystals were grown in 100 mM Hepes pH 7.5, 17% (*w*/*v*) PEG 8000, and had maximum dimensions of 15 × 15 × 200 μm.

Prior to data collection, the crystals were harvested from the drops using mounted cryoloops (Hampton Research), briefly transferred to a cryoprotection buffer consisting of mother liquor supplemented with 20% (*v*/*v*) glycerol, and subsequently flash-cooled and stored in liquid nitrogen. Diffraction data sets were collected to 1.9 Å resolution at beamline ID23-2 at the European Synchrotron Radiation Facility (Grenoble, France) at 100 K using a MAR CCD detector and the helical data collection method as implemented at the beamline [37]. All data sets were processed and scaled using the *XDS/XSCALE* programs [38,39]. The crystals belong to space group P4_1_ with unit cell dimensions of a = 124.5, b = 124.5, c = 77.5 Å and α = β = γ = 90° and contain two molecules in the asymmetric unit. This corresponds to a Matthews’ coefficient of 2.27 Å^3^ Da^−1^, giving a solvent content of ~46%. The data collection and processing statistics are summarized in Table 1.

**Table 1:** Crystallographic statistics

	**Cap32/34**
**Data Collection**	
Space Group	P4_1_
Cell Dimensions	
a, b, c (Å)	124.5, 124.5, 77.5
α, β, γ (º)	90, 90, 90
Resolution Range (Å)	50-2.2 (2.3-2.2)
Number of Reflections	367874
Number of Unique Reflections	60185
Completeness (%)	99.8 (99.6)
Multiplicity	
*R*_*merge*_^†^	14.9 (83.5)
<I/σI>	14.2 (3.8)
**Refinement**	
*R*_*work*_^‡^	0.226
*R*_*free*_^§^	0.265
R.m.s. deviations	
Bond lengths (Å)	0.008
Bond angles (°)	1.36
**Ramachandran Analysis**	
Residues in most favoured regions (%)	95.9
Residues in allowed regions (%)	4.1
Outliers (%)	0
Model statistics	
Protein residues:	
No. in subunit A & B	514
*B*-factor A & B (Å^2^)	15.4
Additional groups:	
Water (No. / *B*-factor)	328 / 37.9

Values in parentheses refer to the highest resolution shell.

^†^*R*_*merge*_ = Σ_*hkl*_*Σ*_*i*_*|*I_*i*_(*hkl*) - < I(*hkl*) > *|/* Σ_*hkl*_*Σ*_*i*_I_*i*_(*hkl*); where I_*i*_(*hkl*) is the intensity of the *i*th measurement of reflection *hkl* and < I(*hkl*) > is the mean value of I_*i*_(*hkl*) for all *i* measurements.

^‡^*R*_*work*_ = Σ_*hkl*_||*F*_*o*_|-|*F*_*c*_||/Σ|*F*_*o*_|*,*where *F*_*o*_ is the observed structure factor and *F*_*c*_ is the calculated structure factor.

^§^*R*_*free*_ is the same as *R*_*cryst*_ except calculated with a subset, 5%, of data that are excluded from refinement calculations.

### Structure solution and refinement

Initial phases were obtained by molecular replacement (MR) using the program *CNS*[40]. The structure of CapZ from *Gallus gallus* (PDB code 1IZN) [32] with the solvent ions and the flexible β-subunit C-terminus (residues 252–277) omitted was used as starting model. The structural model was refined using *CNS*, including rigid body, simulated annealing, energy minimization, and individual B-factor refinement in several cycles. Manual inspection, rebuilding, and addition of water molecules were performed with *Coot*[41]. Analysis of the Ramachandran plot reveals ~96% of the residues in most favourable regions and none in disallowed regions. The accuracy of the protein structure model was validated using *MolProbity*[42]. The final model contains residues 2–272 (and 2–270 for the second molecule within the asymmetric unit, respectively) of the 281 residues of the α-subunit and all residues of the β-subunit except for residues 1, 140–145 and 251–272 (253–272 for the second molecule within the asymmetric unit). The structure was deposited in the Protein Data Bank (PDB code 4AKR). All figures were prepared with *PyMOL*[43]. Structural alignments were conducted using least squares superposition (LSQ) as implemented in *Coot*[41].

## Results and discussion

### Overall structure of *Dictyostelium discoideum* Cap32/34

**Figure 1:**
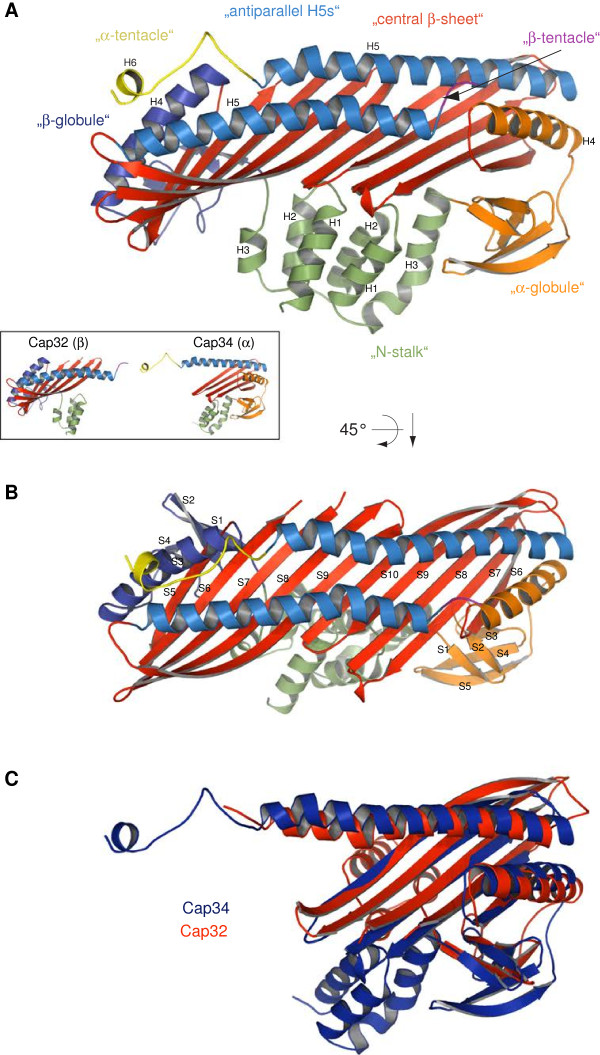
**Crystal structure of *Dictyostelium discoideum *Cap32/34.** A) Ribbon presentation of Cap32/34. The structural motifs are shown in different colours. For clarity and comparability we used the same motif and colour scheme as in [30]. The helices are numbered from the N- to the C-terminus. B) Top view of the structure highlighting the β-strands. Compared to CapZ, one more β-strand could be assigned to both the α-globule and the β-globule region. C) Superposition of Cap32 (red) and Cap34 (blue). While the globule regions are markedly similar, the N-stalk regions point to different directions demonstrating the pseudo 2-fold symmetry of the CP.

Crystals of the Cap32/34 protein were obtained by the hanging-drop vapour diffusion technique. The crystals belong to the tetragonal space group P4_1_ with unit-cell parameters of a = 124.5, b = 124.5, c = 77.5 Å and α = β = γ = 90°, and contain two molecules per asymmetric unit (Table 1). The structure was solved by molecular replacement using the crystal structure of CapZ from *Gallus gallus*[32] as a search model (PDB code 1IZN). The structural model was refined to 2.2 Å resolution with a final *R*_work_ of 22.6% and an *R*_free_ of 26.5% (Figure 1). Superposition of the two Cap32/34 molecules within the asymmetric unit revealed only small deviations in their overall structures, with a root-mean-square deviation (r.m.s.d.) of 0.3 Å for 512 common C_α_ atoms. Equivalently to chicken CapZ [32], the α- and β-subunits of Cap32/34 from *Dictyostelium discoideum* have strikingly similar secondary and tertiary structures (Figure 1C), despite showing only modest homology at the amino acid sequence level. Furthermore, the two subunits are extensively intertwined, resulting in a pseudo 2-fold axis of rotational symmetry of the entire molecule. Given the tight interactions occurring between the CP subunits, it is not surprising that the heterodimer is extremely stable as opposed to the individual subunits. Briefly, Cap32/34 has the shape of a mushroom, comprising a stalk (“N-stalk”) and a cap (“central β-sheet” and “antiparallel H5s”). The mushroom stalk is composed of six anti-parallel α-helices, of which three are contributed from the N-terminus of each subunit (H1–3). Stretches of five antiparallel β-strands of the α-subunit (S1–5) and four of the β-subunit (S1–4) are located next to the stalk and under the cap of the mushroom (“α- and β-globule”). The cap consists of a single ten-stranded antiparallel β-sheet formed by five β-strands of each subunit (S6–10).

### Cap32/34 shows the same overall architecture as CapZ

**Figure 2:**
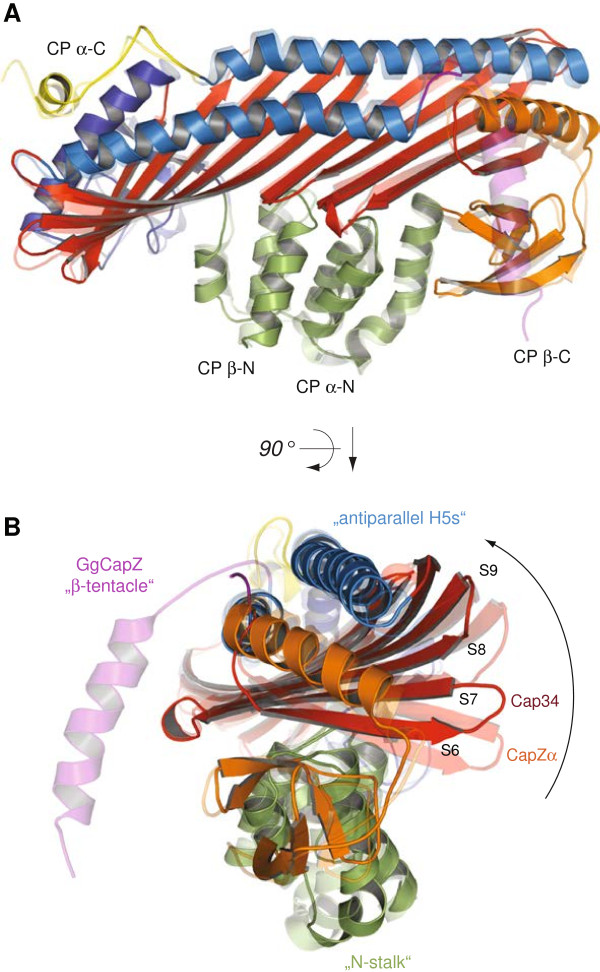
**Structural conservation and conformational flexibility within cytoplasmic and muscle-specific CP.** Orthogonal views of Cap32/34 superposed onto CapZ over the C_α_ positions of the entire CP molecules. Structural motifs are coloured as in Figure 1. To facilitate orientation some domains and structural elements are indicated.

**Figure 3:**
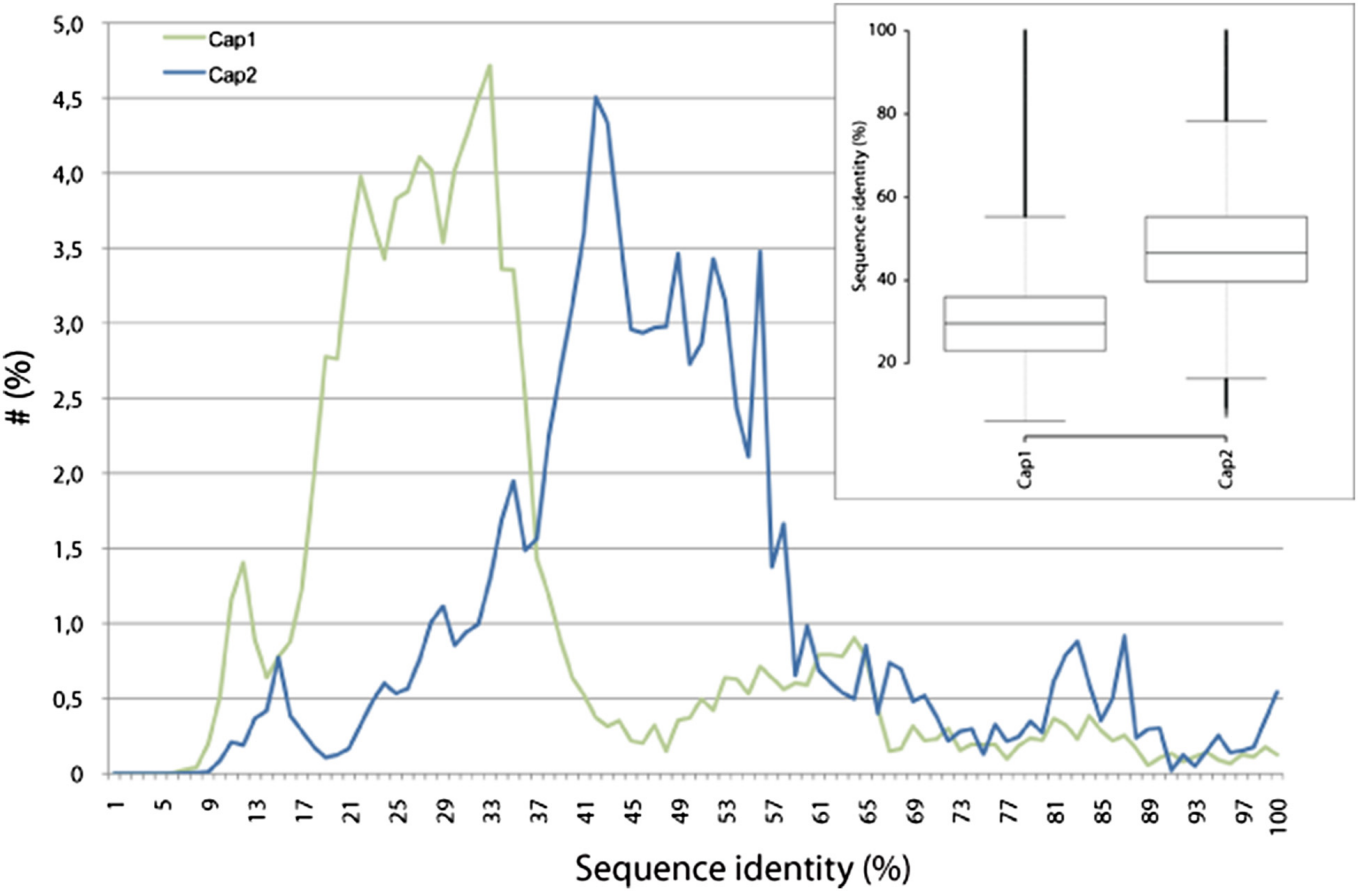
**Sequence identity comparison of CP subunits.** The scores of the sequence identity matrices of the CP subunits were rounded and the percentage of sequences plotted against the sequence identity. The inlet contains box plots of the data for each CP subunit. 368 α-subunit and 299 β-subunit CP sequences were derived from CyMoBase [44,45]. For calculating the sequence identities poorly aligned positions and divergent regions of the alignments were removed using Gblocks [46]. Sequence identity matrices (2D-matrix tables containing sequence identities scores for each pair of sequences) were obtained by calculating the ratio of identities to the length of the longer of the two sequences after positions where both sequences contain a gap were removed.

Superposition of the Cap32/34 molecule onto its homolog CapZ (PDB code 1IZN) resulted in an r.m.s.d. value of ~1.7 Å over 498 equivalent C_α_ atoms (the flexible β-subunit C-termini were excluded), which illustrates the highly conserved architecture of the two CP variants (Figure 2A). While the α-subunits of the two homologs superposed with an r.m.s.d. of ~1.7 Å over the C_α_ atoms (264 residues), the β-subunits match better (r.m.s.d. of ~1.0 Å for 242 residues excluding the β-tentacle), indicative of the latter being structurally more strongly conserved. This is in agreement with findings based on sequence comparisons (Figure 3). In order to quantitatively determine which of the CP subunits is more conserved we calculated sequence identity matrices for all CP subunits in all eukaryotes that have been annotated recently (Hammesfahr and Kollmar, submitted to BMC Evolutionary Biology). Because the data includes sequences from all branches of the eukaryotes each subunit shows a broad distribution. The comparison of the medians of the populations shows that Cap2 (Capβ) is considerably stronger conserved than Cap1 (Capα).

### Cap32/34 reveals strong conformational flexibility in the α-subunit

Comparing the secondary structural elements of Cap32/34 and CapZ, the β-sheets in the globule regions of Cap32/34 each comprise one additional β-strand. The most prominent structural difference is located in the α-subunits in the part of the central β-sheet that is connected to the α-globule and opposite to the β-tentacle (Figure 2B). The loops connecting the β-strands move towards the antiparallel H5s giving Cap32/34 a more compact structure compared to CapZ. Based on the first crystal structure CapZ has been thought to have a fairly rigid structure except for the mobile β-tentacle. Recently and surprisingly, the structure of CapZ in complex with V-1 showed that CapZ consists of two rigid domains that undergo conformational changes but do not correspond to the two subunits [30]. The smaller domain contains the β-globule, some β-strands of the central β-sheet, a small part of the β-H5 helix, and the α-tentacle. The crystal structure of a C-terminal truncation mutant (CapZβΔC) confirmed that CapZ has an intrinsic conformational flexibility within these two domains [30]. The smaller domain contains the region that establishes the initial electrostatic contact with the actin-filament and conformational flexibility might therefore either prevent strong binding or be pivotal for uncapping. Here, Cap32/34 shows a different type of strong conformational flexibility that is located in the α-subunit (Figure 2B). This part is located opposite to the β-tentacle, which establishes the second actin-binding interaction. It might be important for modulating actin-binding through its influence on the tightly connected antiparallel H5 helices to which the β-tentacle is linked. Based on the structure of CapZ bound to the actin filament [31] this region would also be ideally suited for binding CP to the membrane, either directly or mediated by another molecule. Surprisingly, in activated macrophages and platelets CP appears to be simultaneously bound to membranes and actin filaments [47], which would not occur if PIPs bound to the molecule that have an uncapping function. This suggests the possibility that this region of cytoplasmic CP could serve as a binding site for non-PIP lipids in motile cells, thereby mediating membrane attachment of actin. Thus, CP could have an additional role in the dendritic nucleation model apart from capping the barbed end of actin filaments.

### Structure and function of the tentacles

**Figure 4:**
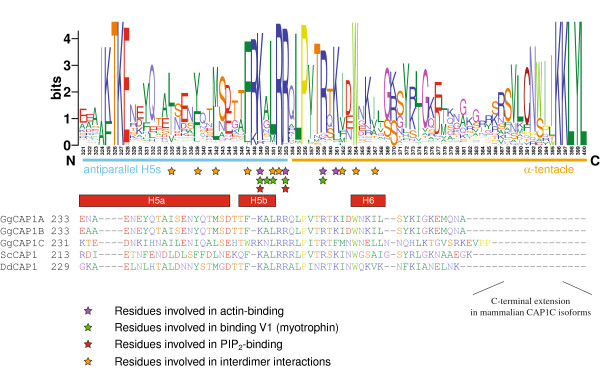
**Sequence conservation within the actin-binding region of the α-subunits.** The sequence logos are based on 368 α-subunit sequences and illustrate the sequence conservation within the multiple sequence alignment of the α-subunits. Here, only the C-termini of the α-subunits are shown because most of the residues implicated in actin binding map to this region (For the representation of the entire α-subunits see Additional file 1). For better orientation, the sequences of five representative α-subunits are shown: the three isoforms of chicken Cap1 for comparison because all previous crystal structures have been obtained from chicken Cap1α, the yeast Cap1 as one of the targets of mutagenesis experiments, and *Dictyostelium* Cap34 whose structure is presented here. Secondary structural elements as determined from the chicken CapZ crystal structure are drawn as yellow arrows (β-strands) and as red boxes (α-helices). Residues important for inter-heterodimer binding, V-1 binding, PIP_2_-binding, and actin-binding are highlighted by orange, green, red, and purple stars, respectively. Numbering below the logos refers to positions in the multiple sequence alignment (The full-length multiple sequence alignment of the α-subunits is available as Additional File 2).

Like in CapZ’s α-subunit the C-terminus of Cap34 includes a short amphipathic α-helix (also called α-tentacle), which is tightly connected by hydrophobic contacts to the body of the β-subunit through a strictly conserved tryptophan residue (Trp-267 in Cap34 from *Dictyostelium discoideum*, Trp-271 in chicken CapZ; Figure 4). The α-tentacle is bound to the β-subunit of CP in all crystal and NMR structures. Especially the NMR analyses show that the flexibility of the α-subunit’s C-terminus is limited to the last 12 residues (L275 – A286 in human Cap1α), which are C-terminal to the strictly conserved tryptophan residue and the 1-turn helix [48,49]. In addition, the C-terminal truncation mutants mouse Cap1α ΔC13 [50] and yeast Cap1α ΔC10 [51] showed only a weak effect on actin binding as did many single residue mutations in the C-terminus of yeast Cap1 [51]. In contrast, longer C-terminal truncations of 28 (mouse Cap1α ΔC28; [50,52]) and 30 residues (yeast Cap1 ΔC30; [51]) abolished actin-binding. In view of the tight and conserved interaction of the antiparallel helices with the central β-sheet the effects of the longer C-terminal truncations could also be due to the disturbance of the structural stability of this region. Thus the α-subunit’s interaction with actin is either solely mediated by the basic patch, in which case the α-tentacle would not move but retain the integrity and stability of the CP dimer, or the α-tentacle moves out of its position to bind actin thus opening a hydrophobic patch on the CP surface. These possibilities can only be tested by mutations that do not disturb the stability of this region. Based on the NMR experiments, the results from the short C-terminal truncations, and the many single residues mutations in the α-tentacle it seems most likely that the α-tentacle is not moving upon actin-binding. The only flexible region consists of the C-terminal 12 residues, which, however, are not strongly conserved and only show a slight effect on actin-binding.

**Figure 5:**
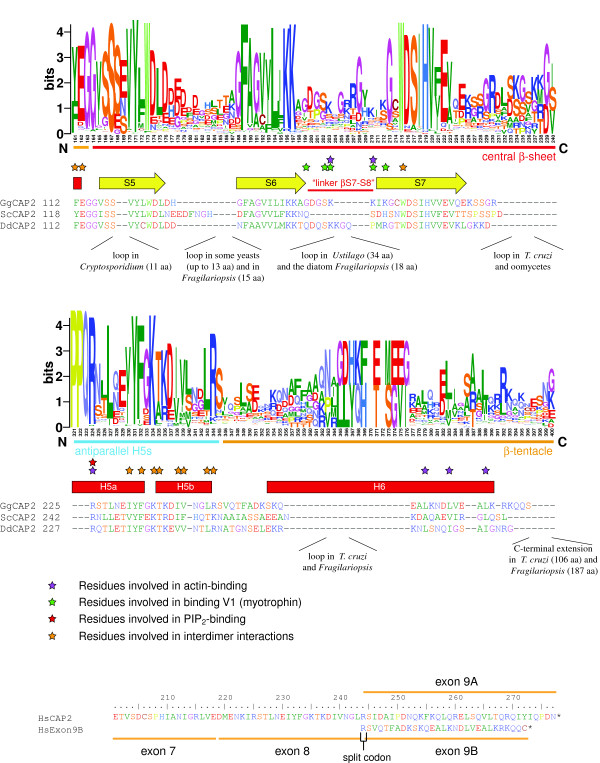
**Sequence conservation within the actin-binding region of the β-subunits.** The sequence logos are based on 299 β-subunit sequences and illustrate the sequence conservation within the multiple sequence alignment of the β-subunits. Two regions known to be important for actin-binding are shown (For the representation of the entire β-subunits see Additional file 3). For better orientation, the sequences of three representative β-subunits are shown: chicken Cap2 of which all previous crystal structures have been obtained, the yeast Cap2 as one of the targets of mutagenesis experiments, and *Dictyostelium* Cap32 whose structure is presented here. Secondary structural elements, important residues indicating various interactions, and taxa/species with elongated loops are denoted as in Figure 4 (The full-length multiple sequence alignment of the β-subunits is available as Additional File 4). Loops, which exist only in single species, have been removed to shorten the alignment by the number of residues as indicated. Numbering below the logos refers to positions in the multiple sequence alignment.

In contrast to the α-tentacle, neither Cap32/34 nor CapZ crystals grown at physiological pH provided an interpretable electron density for the C-terminal segment of the β-subunit (β-tentacle), indicating that this part of the CP molecule is highly mobile. Molecular dynamics studies confirmed the highly flexible nature of this region [53] and NMR experiments showed that the β-tentacle adopts a coil structure in solution [49]. Crystals of native CapZ have previously been soaked into an acidic solution, which stabilized the β-tentacle and allowed its structure to be solved [32]. Hereby it was demonstrated that the β-tentacle also comprises a short amphipathic α-helix, which, more importantly, extends out from the main body of the protein without making any specific interactions with CP. Although the β-tentacle sequence is not conserved in general, the three hydrophobic positions (residues L258, L262, and L266 in *Gg*CapZ) at intervals of four residues are conserved (Figure 5) and exchanging them by polar residues abolishes actin-binding [50]. Therefore, CP has been proposed to bind to actin in two steps—first electrostatically through the basic patch on its α-subunit’s C-terminus, followed by hydrophobic interactions via its amphipathic β-tentacle [11]. The β-tentacles’ helical structure is stabilized in the crystal structure by interaction with a symmetry-related molecule [32]. We also soaked the *Dictyostelium* Cap32/34 crystals in acidic solution but did not see additional electron density in the region where the β-tentacle would be located.

### Structure and flexibility of a linker connecting β-strands of the central β-sheet in the β-subunit

**Figure 6:**
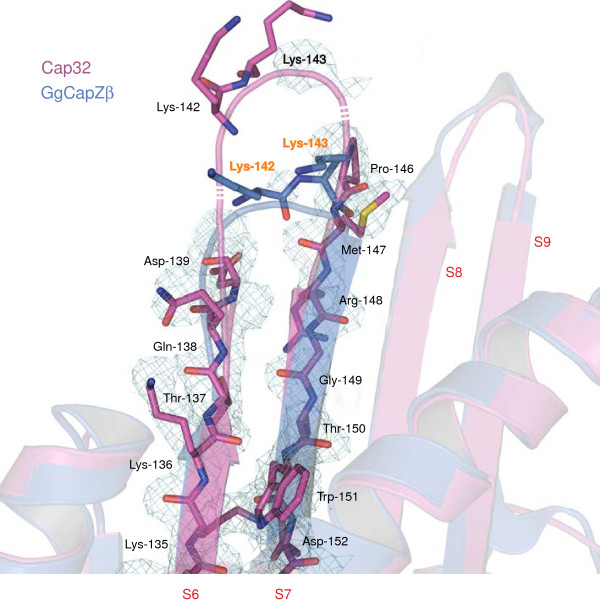
**Structure of “linker βS7–S8” from Cap32.** Ribbon representation of the region around “linker βS7–S8” of the superposed Cap32 and chicken CapZβ structures. The residues of β-strands S7 and S8 of Cap32 and the two lysines K142 and K143 of chicken CapZ are shown as stick models. The part of Cap32 that is not visible in the electron density has been drawn illustrating the hypothetical positions of the lysines K142 and K143 of *Dictyostelium* Cap32 for comparison.

In addition to the region connecting the β-strands of the central β-sheet of the α-subunits opposite to the β-tentacle, the crystal structure of Cap32/34 reveals a notable difference between the β-subunits of the two homologs. Due to disorder no electron density could be assigned to residues Gln-140–Gln-145 of the Cap32 central β-sheet (Figure 6). This region corresponds to a solvent-accessible turn region between S7 and S8 (corresponding to S6 and S7 in chicken Capβ), thus being referred to in this study as “linker βS7–S8” (β denotes the CP β-subunit). Since this segment is well-ordered in the CapZ structure, one could assume that the difference in flexibility might arise from “linker βS7–S8” undergoing conformational dynamics and serving as a binding site in the Cap32 molecule. As can be seen from the sequence alignment (Figure 5), “linker βS7–S8” harbours up to two basic amino acids (Lys-142, Lys-143 both in Cap32 from *Dictyostelium discoideum* and chicken Cap2, respectively), which, based on the crystal structure of CapZ, are positioned directly at the tip of the loop. Since acidic residues are not located in immediate proximity, the molecular surface of this region exhibits a pronounced positive electrostatic potential, making it particularly suitable for electrostatic interactions with negatively charged target sites. The sequence alignment further reveals that Lys-142 and Lys-143 of Cap32 are C-terminally flanked by three additional residues (Gly-144, Gln-145, Pro-146) resulting in an elongated linker region.

### Implication of “linker βS7–S8” from Cap32 in actin-binding

In a recent NMR study of mouse cytoplasmic CP (α1/β2) interacting with the inhibitor proteins CARMIL-1 and V-1, respectively, “linker βS7–S8” was found to undergo significant chemical shift changes, suggesting that this site is involved in actin-binding [48,49]. As part of the same study, charge reversal mutations of Lys-142 and Lys-143 severely decreased the affinity for the barbed end [49]. In contrast, lysine to alanine mutations indicated that the residues Lys-142 and Lys-143 of mouse cytoplasmic CP (α1/β2) hardly affect actin affinity [50]. However, mutations of the arginines and lysines of the “basic triad” already showed that substitutions by alanine only resulted in minor effects in contrast to the severe impact of the charge reversal and double/triple mutations on actin-binding. The so called “basic triad”, three highly conserved basic residues in close proximity to the CP α-subunit C-terminus, is supposed to mediate the initial contact with the barbed end of actin filaments [31]. These residues are exposed to the solvent and thus provide the center of a basic patch on CP. Several basic and conserved residues in close proximity have also been implicated to be involved in actin-binding, namely residues R195, K223, and R225 of the β-subunit of CapZ [50]. The “linker βS7–S8” is also in close vicinity to the “basic triad” but the two lysines are not strictly conserved and even absent in fungi and yeasts (Figure 5). Thus, we suppose that the two lysines of “linker βS7–S8”, similar to the basic residues R195, K223, and R225, are not essential for the major contact with actin, which is mediated by the “basic triad”, but contribute to the basic patch to support barbed end capping on actin-binding. In addition, the “linker βS7–S8” is located directly next to the hinge of the two rigid domains identified in CapZ that undergo conformational changes [30].

### Cap32/34 and lipid-binding

CP is known to be inhibited by polyphosphoinositedes such as phosphatidylinositol 4,5 bisphosphate (PIP_2_) [54]. PIP_2_ does not only bind to CP but is also able to uncap CP from the barbed ends [55]. A structure of PIP_2_ bound to CP is not available yet. It is known, however, from structures of other actin-binding proteins in complex with PIP_2_ or the sugar moieties of PIP_2_ that PIP_2_ preferentially binds to protein-specific patterns of lysines and arginines. Therefore, the region around the “basic triad” that harbours many solvent exposed lysines and arginines has been proposed to be the PIP_2_ binding site of CP. A triple mutation of two of the basic residues of the “basic triad” (K256 and R260) together with a closely located arginine of the β-subunit (R225) has been most effective in abolishing PIP_2_-binding [25]. As in the studies of the interaction of CP with actin, single alanine mutations had been less effective compared to charge reversal, double and triple mutants. However, most of the basic residues around the “basic triad”, including βR195, βR223, βR225, and the two lysines of “linker βS7–S8”, are also conserved in all CP (Figures 4 and 5) and thus could also contribute or be responsible for PIP2 binding. To unambiguously reveal the PIP_2_ binding site a more comprehensive mutational study or a high-resolution structure would be necessary. We also sought to characterize the structure of Cap32/34 in complex with the lipid PIP_2_. To accomplish that, we performed both co-crystallization and crystal soaking experiments in which the molecular ratio of the ligand was varied. Although crystals were obtained by co-crystallization there was no evidence for additional electron density. Similarly, our attempts to bind the inhibitor to Cap32/34 by soaking the crystals were not successful.

### Possible interaction site of CapZ with the Z-discs of skeletal muscles

**Figure 7:**
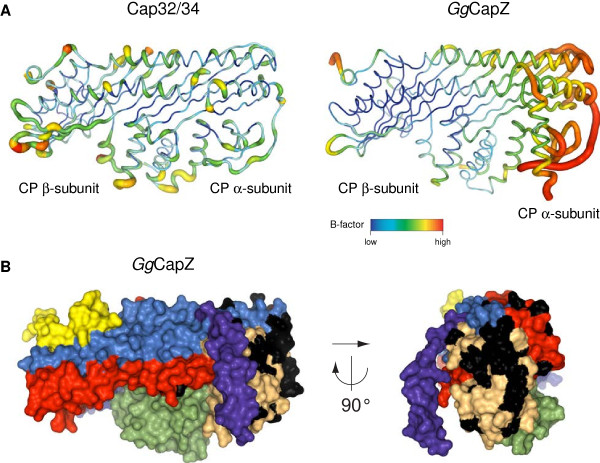
**Putative binding site for Z-disc proteins.A**) Coil representation of Cap32/34 and chicken CapZ illustrating the B-factor distribution of the C_α_ atoms. **B**) Surface presentation of chicken CapZ with structural motifs defined and coloured as in Figure 1. Residues that are different between the α-subunit isoforms Capα1 and Capα2 are highlighted in black. These residues cluster in the same part of the α-globule that shows increased B-factors in the chicken CapZ structure.

Another difference between Cap32/34 and CapZ became apparent when the distribution of B-factor values was compared. As illustrated in Figure 7, the CapZ α- subunit segment spanning from Leu-101–Leu-117 exhibits a substantially higher average B-factor compared to the corresponding region in Cap32/34 (~86.8 Å^2^ for CapZ compared to ~24.2 Å^2^ for Cap32/34). Furthermore, the two homologs do not only adopt markedly different conformations within this part of the molecule (C_α_ r.m.s.d. of ~3.0 Å) but also display different secondary structural elements (residues Lys-103 – Pro-108 of CapZ exhibit a random coil structure, whereas the equivalent region in Cap32/34 is part of a β-strand). CapZ has recently been found to associate with the giant sarcomeric protein nebulin, which is thought to target the protein to the Z-disc [56]. Surprisingly, cytoplasmic CP also binds to nebulin in vitro, whereas in myocytes, which contain both CP variants, only CapZ has been found at the Z-disc [56]. Consequently, another binding partner might be responsible for targeting CapZ to the Z-disc. Based on our observation that the CapZ molecule includes a solvent-accessible region greatly differing in both flexibility and conformation from that of Cap32/34, residues Leu-101 – Leu-117 within the α-subunit may contribute to the interaction with the Z-disc of the sarcomere, either in an indirect manner by being involved in mediating the process or through direct binding. These conclusions are in line with the conformational flexibility of the neighbouring regions of the loops connecting S6 and S7, and S8 and S9 (see above) that also revealed differences between cytoplasmic CP and CapZ.

## Conclusions

We here report the first high resolution structure of a cytoplasmic CP. The overall structure of Cap32/34 from *Dictyostelium discoideum* reveals a similar arrangement as compared to its sarcomeric variant CapZ. Like in CapZ, the individual CP subunits exhibit very similar secondary and tertiary structures despite sharing a very low sequence homology. Moreover, the subunits are extensively intertwined and organized in such a way that the molecule has a pseudo 2-fold axis of rotational symmetry down its center point.

As has been observed in CapZ, the Cap32/34 structure showed the attachment of the α-tentacle to the central β-sheet and the antiparallel H5 helices, and supported the highly flexible nature of the β-tentacle, which is proposed to swing out and bind to actin. By superposition onto CapZ we observed considerably structural flexibility in the α-subunits. In Cap34 the region located opposite to the C-terminal β-tentacle moves towards the antiparallel helices that interconnect the heterodimer leading to a more compact CP structure. This bending motion demonstrates additional flexibility in CP to the two domain twisting attributed mainly to the β-subunit as observed in the structure of CapZ complexed with V-1.

Furthermore, there is evidence that, in terms of cytoplasmic CP, an additional protein segment might be important for mediating high affinity capping of actin filaments. Based on the crystal structure of Cap32/34, the molecule comprises a dynamic loop region located between S7 and S8 within its β-subunit, denoted here as “linker βS7–S8”, which has recently been reported to be important for the association of cytoplasmic CP with actin [48]. This observation is in marked contrast to CapZ, in which the corresponding region has been found to be well ordered [32]. Since “linker βS7–S8” provides a positively charged surface close to the basic patch on CP, it might participate in the initial electrostatic binding to acidic regions on the barbed end of actin filaments.

Finally, to date information about potential interaction sites of CapZ with the Z-disc of the sarcomere is not available. By comparing the structures of the two CP variants, we were able to detect a solvent-exposed region within the CapZ α-subunit (residues Leu-101 – Leu-117 located in the α-globule), greatly differing in both conformation and flexibility from that of Cap32/34. We therefore hypothesize that this protein segment might be involved in the binding of CapZ to the Z-disc in muscle cells.

## Abbreviations

CP: Capping protein; *Dd*: *Dictyostelium discoideum*; *Gg*: *Gallus gallus*; PEG: Polyethylene glycol; r.m.s.d: Root mean square deviation; *Sc*: *Saccharomyces cerevisiae*.

## Competing interests

The authors declare that they have no competing interests.

## Authors’ contributions

MK conceived the project. MF designed the constructs and performed cloning. CE and AG purified the protein and performed crystallization trials. Data collection, processing, and phasing were carried out by MK and CE. CE conducted model building and refinement. MK and CE performed structural and sequence analyses, and wrote the manuscript. All authors read and approved the final manuscript.

## Supplementary Material

Additional file 1 **Conserved residues in the CP α-subunits.** This figure contains the sequence conservation of the entire CP α-subunits including all mutagenesis experiments as described in the legend. Click here for file

Additional file 2 **Sequence alignment of the CP α-subunits.** The file contains the alignment of the sequences of CP α-subunits in fasta-format. The data can also be downloaded from CyMoBase 44, 45. Click here for file

Additional file 3 **Conserved residues in the CP ß-subunits.** This figure contains the sequence conservation of the entire CP ß-subunits including all mutagenesis experiments as described in the legend. Click here for file

Additional file 4 **Sequence alignment of the CP ß-subunits.** The file contains the alignment of the sequences of CP ß-subunits in fasta-format. The data can also be downloaded from CyMoBase 44, 45. Click here for file
