# Supplementary material for: Conservation and divergence between cytoplasmic and muscle-specific actin capping proteins: insights from the crystal structure of cytoplasmic Cap32/34 from Dictyostelium discoideum
Source: BMC Struct Biol. 2012 Jun 1;12:12. doi: 10.1186/1472-6807-12-12 (PMC3472329; doi:10.1186/1472-6807-12-12)
Supplement: Additional file 1 — Conserved residues in the CP α-subunits. This figure contains the sequence conservation of the entire CP α-subunits including all mutagenesis experiments as described in the legend. [file 1472-6807-12-12-S1.pdf]

Sequence conservation within the actin-binding region of the α-subunits

The sequence logos illustrate the sequence conservation within the multiple sequence alignment of the α-subunits. For better orientation, the sequences of five representative α-subunits are shown: the three isoforms of chicken Cap1 for comparison because all previous crystal structure have been obtained from chicken Cap$\alpha 1$, the yeast Cap1 as one of the targets of mutagenesis experiments, and *Dictyostelium* Cap34 whose structure is presented here. Secondary structural elements as determined from the chicken CapZ crystal structure are drawn as yellow arrows (β-strands) and red boxes (α-helices). Residues important for inter-heterodimer binding, V-1 binding, CARMIL binding, PIP$_2$-binding, and actin-binding are highlighted by orange, green, light-blue, red, and purple stars, respectively (see legend below). The sequence logos are based on 368 α-subunit sequences. Coloured dots point to the respective study in which the designated mutations have been analysed (for details see legend below). Amino acid numbering of the mutations refers to the sequence of the respective species that had been analysed. Chicken and mouse numberings are identical. Dark and light coloured boxes around the mutations indicate whether the mutation had a strong or weak effect on the respective studied interaction. Those taxa and species are mentioned that caused the gaps in the alignment. If numbers for the loop-lengths are given then these numbers of positions have been removed from the alignment for compaction and clarity.

Effect

strong weak

Residues involved in actin-binding

Residues involved in binding V1 (myotrophin)

Residues involved in PIP$_2$-binding

Residues involved in binding the CP-binding motif of CARMIL proteins

Residues involved in interdimer interactions

Chicken CP ($\alpha 1\beta 1$): PIP$_2$-binding and actin-binding [K. Kim *et al.* J Biol Chem **2007**, *282*, 5871-5879]

Yeast CP: actin-binding [K. Kim *et al.* J Cell Biol **2004**, *164*, 567-580]

Chicken CP ($\alpha 1\beta 1$): actin-binding [M.A. Wear *et al.* Curr Biol **2003**, *13*, 1531-1537]

Chicken CP ($\alpha 1\beta 1$): actin-binding [A. Narita *et al.* EMBO J **2006**, *25*, 5626-5633]

Chicken CP ($\alpha 1\beta 1$): V-1 binding and CARMIL binding [S. Takeda *et al.* PLoS Biology **2010**, *8*, e1000416]

Mouse CP ($\alpha 1\beta 2$): actin-binding [T. Kim *et al.* J Mol Biol **2010**, *404*, 794-802]

Mouse CP ($\alpha 1\beta 2$): V-1 binding and actin-binding [A. Zwolak *et al.* J Biol Chem **2010**, *285*, 25767-25781]

Mouse CP ($\alpha 1\beta 2$): CARMIL binding and actin-binding [A. Zwolak *et al.* J Biol Chem **2010**, *285*, 29014-29026]

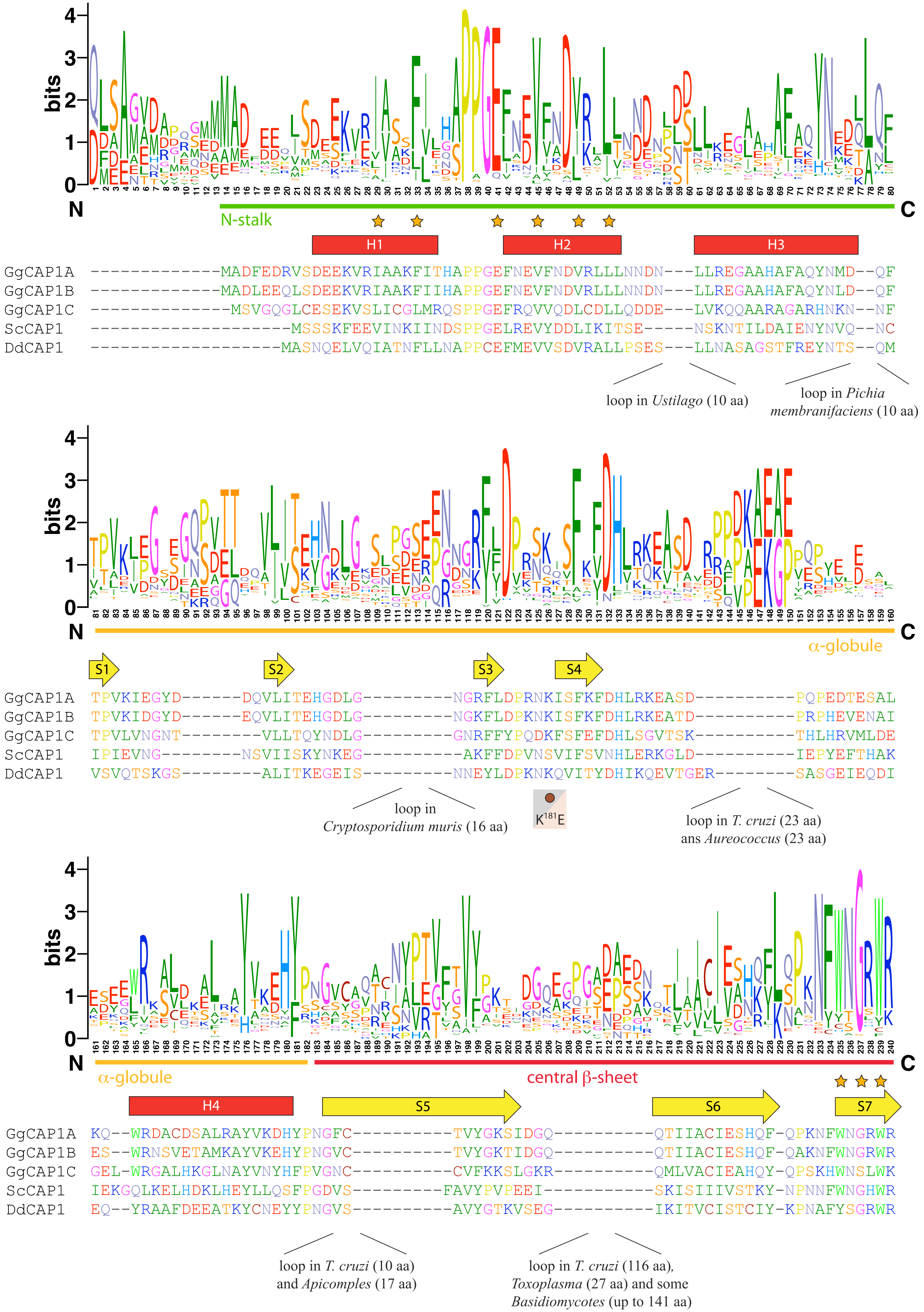
bits
N
C
N-stalk
H1
H2
H3
GgCAP1A -------------MADFEDRVSDEEKVRIAAKFITHAPPGEFNEVFNDVRLLLNNDN---LLREGAAHAFAQYNMD--QF
GgCAP1B -------------MADLEEQLSDEEKVRIAAKFIIHAPPGEFNEVFNDVRLLLNNDN---LLREGAAHAFAQYNLD--QF
GgCAP1C --------------MSVGQGLCESEKVSLICGLMRQSPPGEFRQVVQDLCDLLQDDE---LVKQQAARAGARHNKN--NF
ScCAP1 -------------------MSSSKFEEVINKIINDSPPGELREVYDDLIKITSE-----NSKNTILDAIENYNVQ--NC
DdCAP1 ------------------MASNQELVQIATNFLLNAPPCEFMEVVSDVRALLPSES---LLNASAGSTFREYNTS--QM
loop in *Ustilago* (10 aa)
loop in *Pichia membranifaciens* (10 aa)
bits
N
C
α-globule
S1
S2
S3
S4
GgCAP1A TPVKIEGYD------DQVLITEHGDLG---------NGRFLDPRNKISFKFDHLRKEASD----------PQPEDTESAL
GgCAP1B TPVKIDGYD------EQVLITEHGDLG---------NGKFLDPKNKISFKFDHLRKEATD----------PRPHEVENAI
GgCAP1C TPVLVNGNT--------VLLTQYNDLG---------GNRFFYPQDKFSFEFDHLSGVTSK----------THLHRVMLDE
ScCAP1 IPIEVNG--------NSVIISKYNKEG----------AKFFDPVNSVIFSVNHLERKGLD----------IEPYEFTHAK
DdCAP1 VSVQTSKGS--------ALITKEGEIS---------NNEYLDPKNKQVITYDHIKQEVTGER--------SASGEIEQDI
loop in *Cryptosporidium muris* (16 aa)
K[181]E
loop in *T. cruzi* (23 aa) ans *Aureococcus* (23 aa)
bits
N
C
α-globule
central β-sheet
H4
S5
S6
S7
GgCAP1A KQ--WRDACDSALRAYVKDHYPNGFC----------TVYGKSIDGQ----------QTIIACIESHQF-QPKNFWNGRWR
GgCAP1B ES--WRNSVETAMKAYVKEHYPNGVC----------TVYGKTIDGQ----------QTIIACIESHQF-QAKNFWNGRWR
GgCAP1C GEL-WRGALHKGLNAYVNYHFPVGNC----------CVFKKSLGKR----------QMLVACIEAHQY-QPSKHWNSLWK
ScCAP1 IEKGQLKELHDKLHEYLLQSFPGDVS---------FAVYPVPEEI-----------SKISIIIVSTKY-NPNNFWNGHWR
DdCAP1 EQ--YRAAFDEEATKYCNEYYPNGVS----------AVYGTKVSEG----------IKITVCISTCIY-KPNAFYSGRWR
loop in *T. cruzi* (10 aa) and *Apicomples* (17 aa)
loop in *T. cruzi* (116 aa), *Toxoplasma* (27 aa) and some *Basidiomycotes* (up to 141 aa)

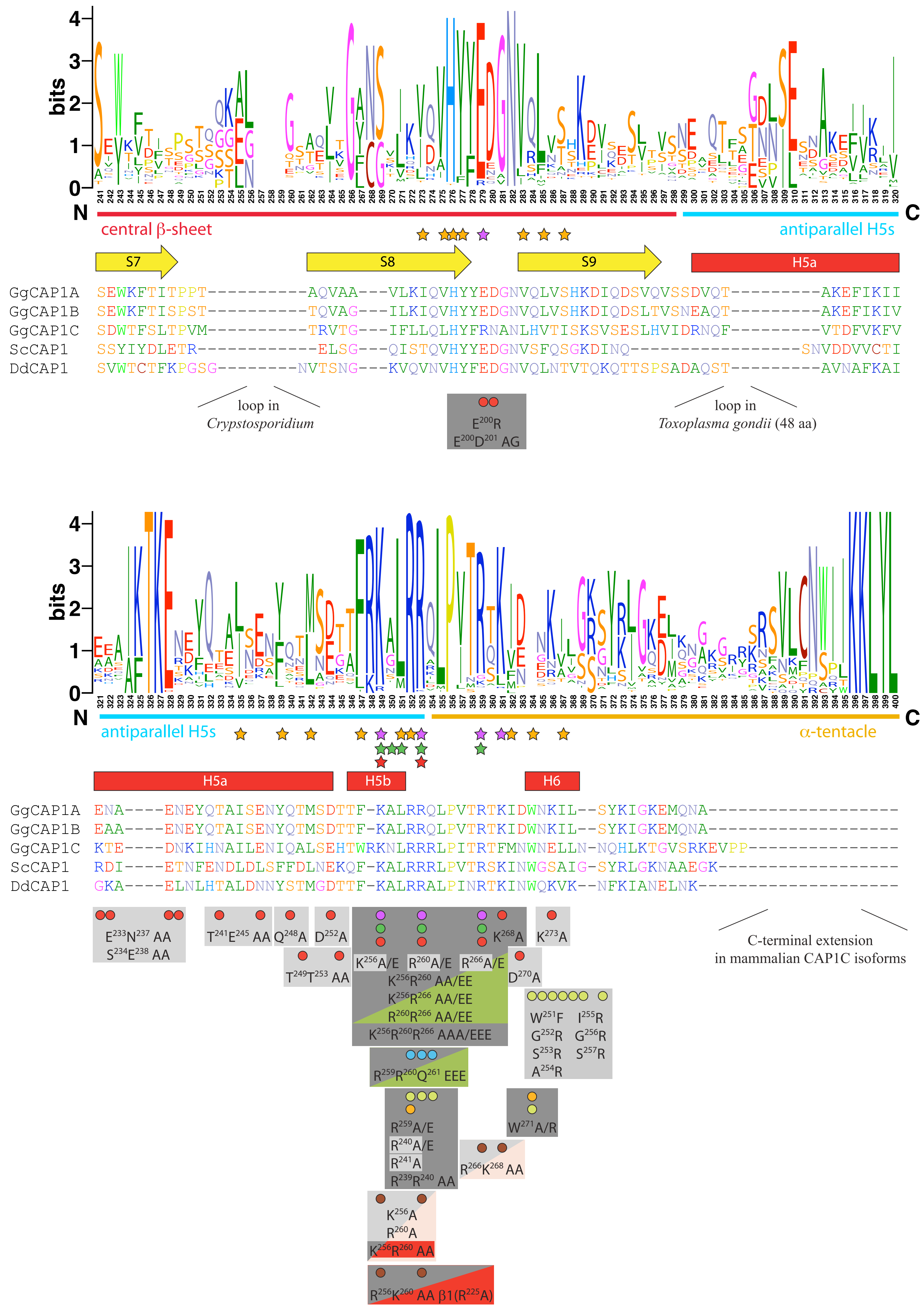

bits
N
C
central β-sheet
antiparallel H5s
S7
S8
S9
H5a
GgCAP1A SEWKFTITPPT----------AQVAA---VLKIQVHYYEDGNVQLVSHKDIQDSVQVSSDVQT---------AKEFIKII
GgCAP1B SEWKFTISPST----------TQVAG---ILKIQVHYYEDGNVQLVSHKDIQDSLTVSNEAQT---------AKEFIKIV
GgCAP1C SDWTFSLTPVM----------TRVTG---IFLLQLHYFRNANLHVTISKSVSESLHVIDRNQF---------VTDFVKFV
ScCAP1 SSYIYDLETR-------------ELSG---QISTQVHYYEDGNVSFQSGKDINQ-----------------SNVDDVVCTI
DdCAP1 SVWTCTFKPGSG--------NVTSNG---KVQVNVHYFEDGNVQLNTVTQKQTTSPSADAQST---------AVNAFKAI
loop in Crypstosporidium
E200R
E200D201 AG
loop in Toxoplasma gondii (48 aa)
bits
N
C
antiparallel H5s
α-tentacle
H5a
H5b
H6
GgCAP1A ENA----ENEYQTAISENYQTMSDTTF-KALRRQLPVTRTKIDWNKIL--SYKIGKEMQNA------------------
GgCAP1B EAA----ENEYQTAISENYQTMSDTTF-KALRRQLPVTRTKIDWNKIL--SYKIGKEMQNA------------------
GgCAP1C KTE----DNKIHNAILENIQALSEHTWRKNLRRRLPITRTFMNWNELLN-NQHLKTGVSRKEVPP--------------
ScCAP1 RDI----ETNFENDLDLSFFDLNEKQF-KALRRRLPVTRSKINWGSAIG-SYRLGKNAAEGK-----------------
DdCAP1 GKA----ELNLHTALDNNYSTMGDTTF-KALRRALPINRTKINWQKVK--NFKIANELNK-------------------
E233N237 AA
S234E238 AA
T241E245 AA
Q248A
D252A
T249T253 AA
K256A/E
R260A/E
R266A/E
K268A
K273A
D270A
K256R260 AA/EE
K256R266 AA/EE
R260R266 AA/EE
K256R260R266 AAA/EEE
W251F
I255R
G252R
G256R
S253R
S257R
A254R
R259R260Q261 EEE
R259A/E
R240A/E
R241A
R239R240 AA
W271A/R
R266K268 AA
K256A
R260A
K256R260 AA
R256K260 AA β1(R225A)
C-terminal extension in mammalian CAP1C isoforms
