# Supplementary material for: Conservation and divergence between cytoplasmic and muscle-specific actin capping proteins: insights from the crystal structure of cytoplasmic Cap32/34 from Dictyostelium discoideum
Source: BMC Struct Biol. 2012 Jun 1;12:12. doi: 10.1186/1472-6807-12-12 (PMC3472329; doi:10.1186/1472-6807-12-12)
Supplement: Additional file 3 — Conserved residues in the CP ß-subunits. This figure contains the sequence conservation of the entire CP ß-subunits including all mutagenesis experiments as described in the legend. [file 1472-6807-12-12-S3.pdf]

Sequence conservation within the actin-binding region of the β-subunits

The sequence logos illustrate the sequence conservation within the multiple sequence alignment of the β-subunits. For better orientation, the sequences of three representative β-subunits are shown: the isoforms of chicken Cap2 for comparison because all previous crystal structure have been obtained from chicken Capβ, the yeast Cap1 as one of the targets of mutagenesis experiments, and *Dictyostelium* Cap32 whose structure is presented here. Secondary structural elements as determined from the chicken CapZ crystal structure are drawn as yellow arrows (β-strands) and red boxes (α-helices). Residues important for inter-heterodimer binding, V-1 binding, CARMIL binding, PIP$_2$-binding, and actin-binding are highlighted by orange, green, light-blue, red, and purple stars, respectively (see legend below). The sequence logos are based on 299 β-subunit sequences. Coloured dots point to the respective study in which the designated mutations have been analysed (for details see legend below). Amino acid numbering of the mutations refers to the sequence of the respective species that had been analysed. Chicken and mouse numberings are identical. Dark and light coloured boxes around the mutations indicate whether the mutation had a strong or weak effect on the respective studied interaction. Those taxa and species are mentioned that caused the gaps in the alignment. If numbers for the loop-lengths are given then these numbers of positions have been removed from the alignment for compaction and clarity.

Effect

strong weak

- Residues involved in actin-binding
- Residues involved in binding V1 (myotrophin)
- Residues involved in PIP$_2$-binding
- Residues involved in binding the CP-binding motif of CARMIL proteins
- Residues involved in interdimer interactions
- *Chicken CP (α1β1): PIP$_2$-binding and actin-binding* [K. Kim *et al.* J Biol Chem **2007**, *282*, 5871-5879]
- *Yeast CP: actin-binding* [K. Kim *et al.* J Cell Biol **2004**, *164*, 567-580]
- *Chicken CP (α1β1): actin-binding* [M.A. Wear *et al.* Curr Biol **2003**, *13*, 1531-1537]
- *Chicken CP (α1β1): actin-binding* [A. Narita *et al.* EMBO J **2006**, *25*, 5626-5633]
- *Chicken CP (α1β1): V-1 binding and CARMIL binding* [S. Takeda *et al.* PLoS Biology **2010**, *8*, e1000416]
- *Mouse CP (α1β2): actin-binding* [T. Kim *et al.* J Mol Biol **2010**, *404*, 794-802]
- *Mouse CP (α1β2): V-1 binding and actin-binding* [A. Zwolak *et al.* J Biol Chem **2010**, *285*, 25767-25781]
- *Mouse CP (α1β2): CARMIL binding and actin-binding* [A. Zwolak *et al.* J Biol Chem **2010**, *285*, 29014-29026]

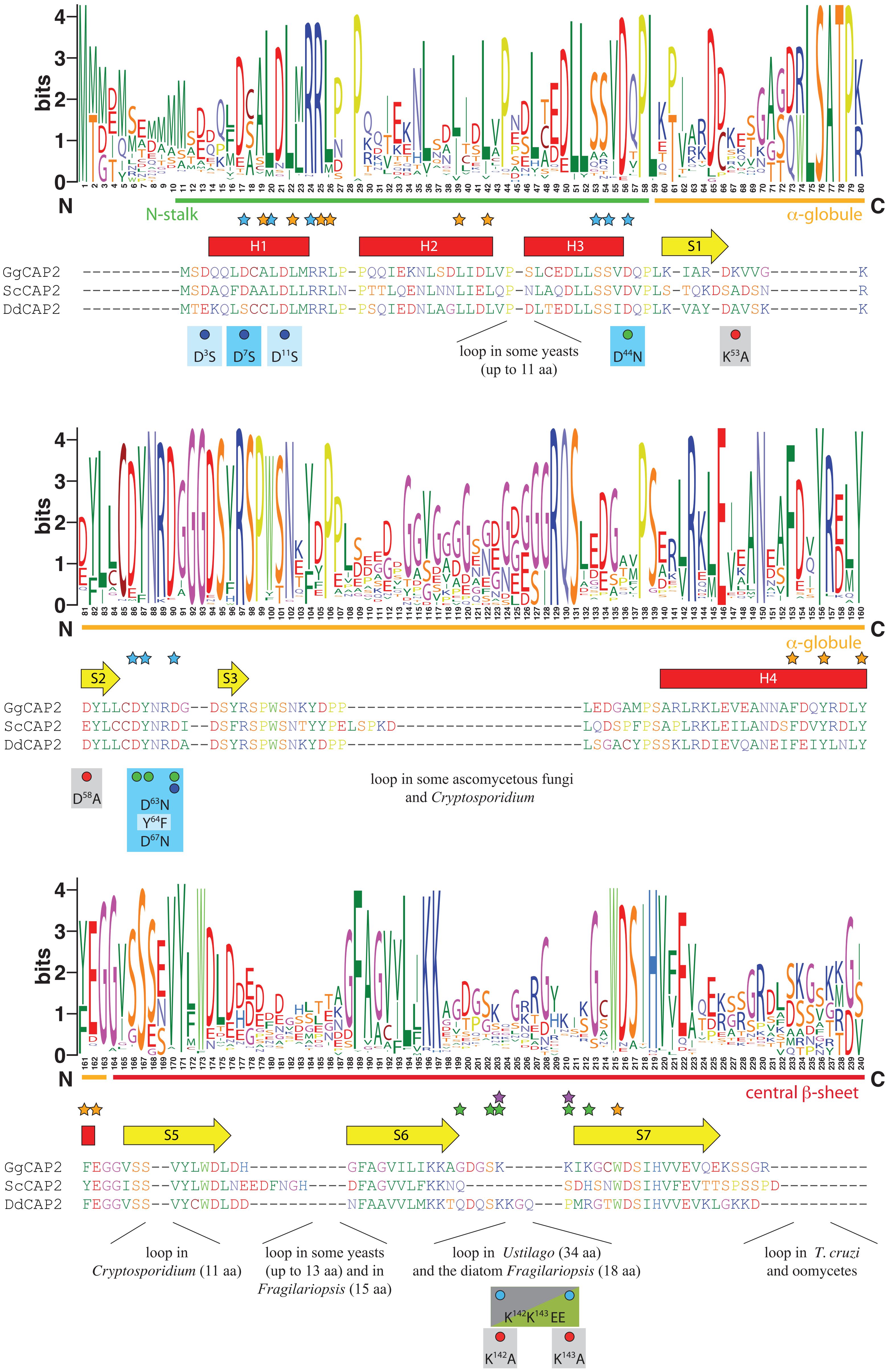

bits
N
C
N-stalk
α-globule
H1
H2
H3
S1
GgCAP2 ----------MSDQQLDCALDLMRRLP-PQQIEKNLSDLIDLVP-SLCEDLLSSVDQPLK-IAR-DKVVG---------K
ScCAP2 ----------MSDAQFDAALDLLRRLN-PTTLQENLNNLIELQP-NLAQDLLSSVDVPLS-TQKDSADSN---------R
DdCAP2 ----------MTEKQLSCCLDLMRRLP-PSQIEDNLAGLLDLVP-DLTEDLLSSIDQPLK-VAY-DAVSK---------K
D^{3}S
D^{7}S
D^{11}S
loop in some yeasts (up to 11 aa)
D^{44}N
K^{53}A
bits
N
C
α-globule
S2
S3
H4
GgCAP2 DYLLCDYNRDG--DSYRSPWSNKYDPP------------------------LEDGAMPSARLRKLEVEANNAFDQYRDLY
ScCAP2 EYLCCDYNRDI--DSFRSPWSNTYYPELSPKD-------------------LQDSPFPSAPLRKLEILANDSFDVYRDLY
DdCAP2 DYLLCDYNRDA--DSYRSPWSNKYDPP------------------------LSGACYPSSKLRDIEVQANEIFEIYLNLY
D^{58}A
D^{63}N
Y^{64}F
D^{67}N
loop in some ascomycetous fungi and *Cryptosporidium*
bits
N
C
central β-sheet
S5
S6
S7
GgCAP2 FEGGVSS--VYLWDLDH----------GFAGVILIKKAGDGSK------KIKGCWDSIHVVEVQEKSSGR----------
ScCAP2 YEGGISS--VYLWDLNEEDFNGH----DFAGVVLFKKNQ----------SDHSNWDSIHVFEVTTSPSSPD---------
DdCAP2 FEGGVSS--VYCWDLDD----------NFAAVVLMKKTQDQSKKGQ---PMRGTWDSIHVVEVKLGKKD-----------
loop in *Cryptosporidium* (11 aa)
loop in some yeasts (up to 13 aa) and in *Fragilariopsis* (15 aa)
loop in *Ustilago* (34 aa) and the diatom *Fragilariopsis* (18 aa)
loop in *T. cruzi* and oomycetes
K^{142}K^{143}EE
K^{142}A
K^{143}A

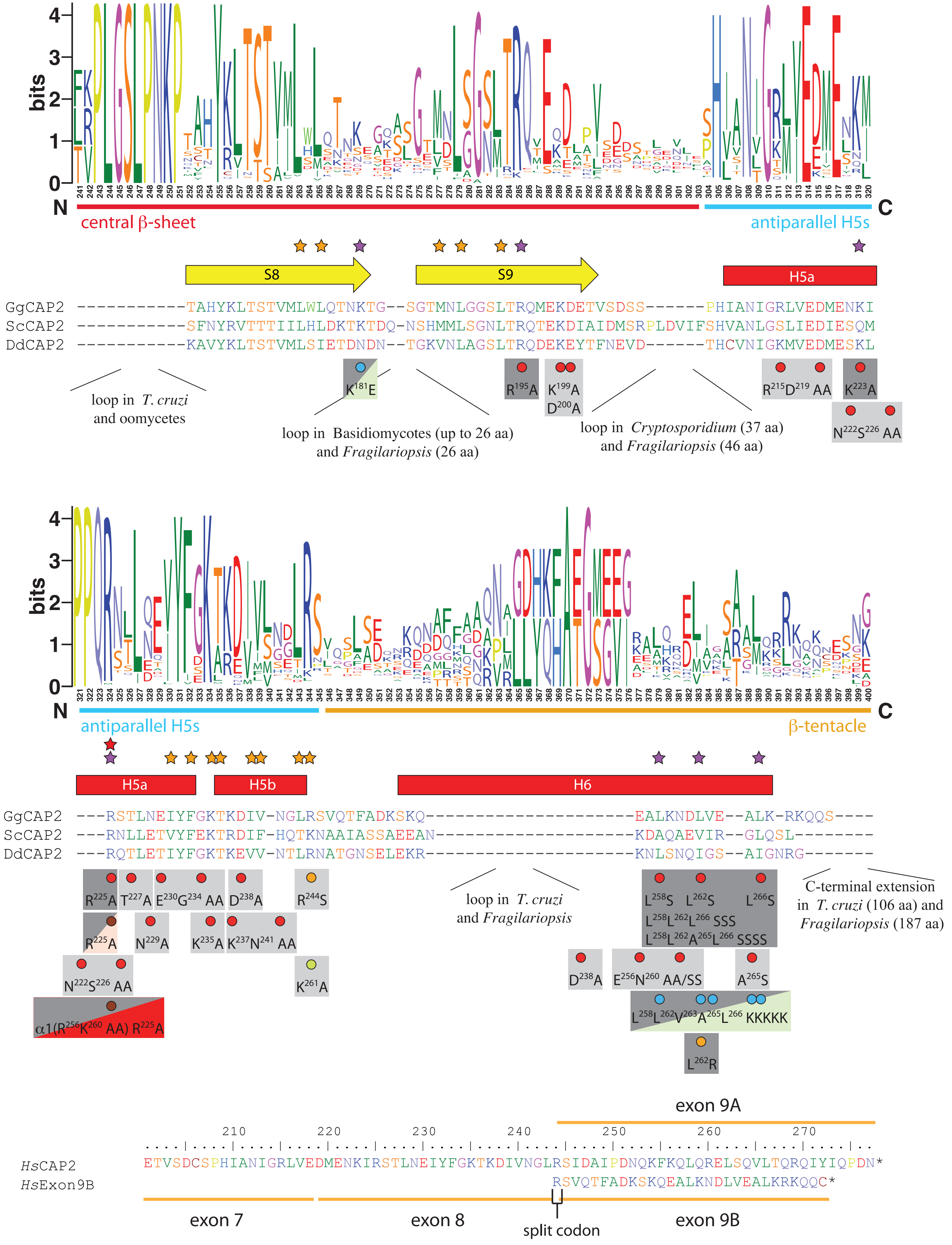
bits
N
C
central β-sheet
antiparallel H5s
S8
S9
H5a
GgCAP2 -----------TAHYKLTSTVMLWLQTNKTG--SGTMNLGGSLTRQMEKDETVSDSS------PHIANIGRLVEDMENKI
ScCAP2 -----------SFNYRVTTTIILHLDKTKTDQ-NSHMMLSGNLTRQTEKDIAIDMSRPLDVIFSHVANLGSLIEDIESQM
DdCAP2 -----------KAVYKLTSTVMLSIETDNDN--TGKVNLAGSLTRQDEKEYTFNEVD------THCVNIGKMVEDMESKL
K181E
R195A
K199A
D200A
R215D219 AA
K223A
N222S226 AA
loop in T. cruzi and oomycetes
loop in Basidiomycotes (up to 26 aa) and Fragilariopsis (26 aa)
loop in Cryptosporidium (37 aa) and Fragilariopsis (46 aa)
bits
N
C
antiparallel H5s
β-tentacle
H5a
H5b
H6
GgCAP2 ---RSTLNEIYFGKTKDIV-NGLRSVQTFADKSKQ--------------------EALKNDLVE--ALK-RKQQS----
ScCAP2 ---RNLLETVYFEKTRDIF-HQTKNAAIASSAEEAN--------------------KDAQAEVIR--GLQSL--------
DdCAP2 ---RQTLETIYFGKTKEVV-NTLRNATGNSELEKR--------------------KNLSNQIGS--AIGNRG-------
R225A T227A E230G234 AA D238A R244S
R225A N229A K235A K237N241 AA
N222S226 AA K261A
α1(R256K260 AA) R225A
loop in T. cruzi and Fragilariopsis
L258S L262S L266S
L258L262L266 SSS
L258L262A265L266 SSSS
D238A E256N260 AA/SS A265S
L258L262V263A265L266 KKKKK
L262R
C-terminal extension in T. cruzi (106 aa) and Fragilariopsis (187 aa)
exon 9A
210 220 230 240 250 260 270
HsCAP2 ETVSDCSPHIANIGRLVEDMENKIRSTLNEIYFGKTKDIVNGLRSIDAIPDNQKFKQLQRELSQVLTQRQIYIQPDN*
HsExon9B RSVQTFADKSKQEALKNDLVEALKRKQQC*
exon 7
exon 8
split codon
exon 9B
